# Scientia machina: a proposed conceptual framework for a technology-accelerated system of biomedical science

**DOI:** 10.3389/fsysb.2025.1576989

**Published:** 2025-03-13

**Authors:** Sean T. Manion

**Affiliations:** ^1^ Psychology Department, Duquesne University, Pittsburgh, PA, United States; ^2^ Science & Evidence Division, AI MINDSystems Foundation, Washington, DC, United States

**Keywords:** systems, science, biomedicine, blockchain, artificial intelligence

## 1 Introduction

What is the brain that it can understand science?

What is science that it can understand the brain?

These two basic questions (with homage to Warren McCulloch in the framing) have guided my career, in which I aimed to understand the brain and to understand science. This journey has taken me from academic lab work to clinical research oversight and government policy to the emerging health and science technology industry and back to academia. It has now led me to co-lead, along with Dr. Jennifer Lovejoy of the Institute of Systems Biology, this section of Frontiers in Systems Biology–Systems Concepts, Theory and Policy in Biology and Medicine. Our journal Chief Editor, Dr. Yoram Vodovotz, has laid out the overarching vision for this and the other sections (Vodovotz, 2021). This Grand Challenge is an effort to add another layer of detail to our Systems Concepts section (Lovejoy, 2024).

Systems biology and systems medicine have roots going back to at least World War II, when biologists and physiologists were recruited into the war effort in the United States and Britain, trained in computational approaches, and joined with engineers and mathematicians to solve complex problems with communications, radar, anti-aircraft guns, and more (Churchill, 1949). This alignment led to the foundation of the field of cybernetics and the related Macy Conferences in the U.S. post-war, while in Britain, “This coalescing of biological, engineering, and mathematics frameworks would continue to great effect a few years later as the Ratio Club” (Husbands and Holland, 2008). In the decades that followed, this robust milieu of ideas would foster the development of everything from general systems theory and information theory to artificial intelligence (AI) and cognitive science (Pickering, 2010). Despite this early alignment, it would be decades before systems biology and systems medicine arose as formal fields of inquiry (Green, 2017).

## 2 Biomedical science as a system

Science has arguably been the most effective way of generating and validating new knowledge for the past few centuries. New technologies and computing approaches now provide us with novel tools to accelerate this process. While early work is being done to explore the use of these new tools for science, the success of real-world applications for detailed aspects of biology and medicine have been limited (McCoy et al., 2024). A comprehensive conceptual framework may be a more effective way to realize the value of technology in accelerating science. Modern science is not a simple holistic process but an amalgam of processes and interests that have accumulated over centuries.

By analyzing this system of science, we can better synthesize a new approach to using the array of emerging technologies now available. This will require us to revisit the current human and institutional processes that govern the creation of new scientific knowledge. A hybrid human-machine approach, aligned with governance in the classic cybernetic style (i.e., control and communication in humans and machines), may allow us to optimize our scientific efforts and advance knowledge for the betterment of all of humanity.

## 3 Knowledge machine

There has been much excitement about the potential of emerging technologies applied to science in recent years—from AI to applications of blockchain technologies and web3 applied as decentralized science (DeSci) (Weidener and Spreckelen, 2024). In these nascent efforts, there has often been an oversimplification of science in order to capture technical requirements to automate or simulate biomedical research.

Science is not done by a single person or organization. Science embodies the contribution of multiple individuals—whose brains are themselves collections of dozens of subsystems (Kirby et al., 2024)—processed through a series of refinement and testing. The results of these are moved through a longitudinal process of validation, contextual framing against prior accumulated knowledge, and consensus determination of evidence level and confidence in the results. Only then does this new knowledge contribute to the body of generalized knowledge we apply to the real world.

Creating a new technology-accelerated knowledge system for biomedical science—what I’m calling here *Scientia Machina*—may be best approached through first articulating the conceptual and epistemological framework of the current system of biomedical science as it moves from data to information to evidence to knowledge and its application. Along the way it passes through layers of trust and is eventually captured in the artifacts of biomedical science we have come to rely on and expect. For applications of emerging technology—such as the automated complex information processing of AI and the automated trust and governance of blockchain—to be most beneficial to science, we should use them to systematically augment and accelerate these processes and create the artifacts of science while maintaining or improving the basic conceptual framework of biomedical knowledge discovery and implementation. Eventually parts of the current system may be sundowned, leading to an even greater acceleration of science.

## 4 Layers of trust

**FIGURE 1:**
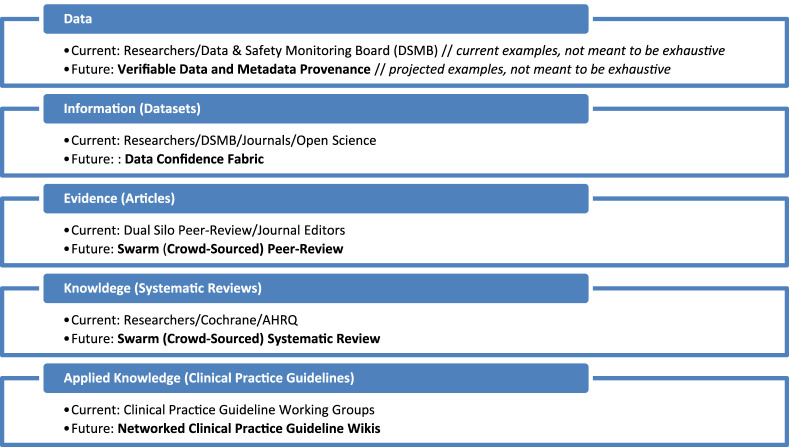
Scientia machina framework—layers of trust in biomedical science.

This *Scientia Machina* framework starts with identifying key layers of trust in the biomedical bench-to-bedside process of evidence-based medicine. Here I have proposed five layers of trust, along with examples of their current artifacts and processes, plus potential approaches to augmenting these with technology and related adjustments to the current workflow (Figure 1).

Data Layer–Data are collected in an experimental and/or clinical context, often based on specific methodology. The principal investigator (PI) and team, along with the equipment and techniques used, are trusted to produce and capture explainable and reproducible data. This layer is only sometimes made transparent and rarely validated.

Future of Data–Data are verifiable through trackable provenance and alignment with related meta-data (e.g., demographics, treatment delivery details, device and equipment specifications, etc.). Data can be accessed for querying and algorithm training without moving, copying, or exposing the data.

Information Layer–Data are combined in datasets with contextual meta-data (e.g., demographics of research participants). The PI and team are trusted to compile, store, and manage this data. It is increasingly becoming requested by funders and publishers to be made available. Some programs promote dataset sharing through centralized repositories or direct PI-to-PI contact.

Future of Information–Data confidence fabrics allow sorting combined datasets based on confidence levels for each data point related to their associated metadata, with deployable programming to temporarily convert non-standard data into a calculable or trainable standard.

Evidence Layer–Analysis of the datasets and testing hypotheses produces results that are interpreted as findings. These are presented as novel assertions, backed by the data and methods, and put into the context of previously identified findings in the field in the form of a manuscript submitted for peer-review. The journal editors and peer reviewers are trusted to confirm the assertions are supported by the evidence and support (or convincingly contradict) previously established knowledge in the field.

Future of Evidence–Swarm approach, i.e., networked, auditable crowdsourcing, to peer review with a wider array of contributors with inputs weighted based on preset governance and continuous crowd feedback for nearer to real-time review with broader, multi-discipline input.

Knowledge Layer–Combined sets of published articles are reviewed by a group of experts against certain criteria to answer specific questions about the state of evidence in the field as systematic reviews and meta-analyses to provide the most up-to-date knowledge in the specific area of focus. The groups of authors, along with editors and peer reviewers of those systematic reviews and meta-analyses, are trusted to have executed and validated, respectively, a thorough and sound assessment of the evidence for the area in question to provide new knowledge.

Future of Knowledge–Swarm approach (see above) to systematic review with network on demand request for new or updated reviews of existing evidence along with evidence threshold signals (i.e., sufficient new evidence in a particular areas prompts new or updated systematic review).

Applied Knowledge Layer–Applications of knowledge can come in various forms, including pharmaceuticals, devices, and procedures. The application of knowledge is periodically assessed for incorporation into clinical practice guidelines (CPG) and similar clinical guidance documents. The CPG group is trusted to have found and appropriately graded all of the available evidence and refined knowledge on a topic area to best inform clinicians on how to address the area optimally.

Future of Knowledge Application–Networked clinical practice guideline wiki (collaboratively edited living document) allowing for continuous, network-refereed input and update of new knowledge.

Each of these layers and their future states can be augmented, enhanced, accelerated, and potentially replaced with appropriate applications of an array of automated processing and trust technologies. Additional administrative areas of biomedical research, such as gap analysis, funding, regulatory review, and more, can be similarly improved.

## 5 The grand challenge

The call to action for this Grand Challenge is to:

a) Consider the core elements of what we need to maintain and continue to elevate from our past and current successful biomedical research and knowledge translation effort, along with areas where those efforts have been flawed, corrupt, or unsuccessful.
b) Critique (and adjust or replace as needed) the Scientia Machina framework proposed here as the backbone for the layers of trust that are the core elements to be maintained as we continue to bring new technologies into biomedical research to accelerate and improve science.
c) Capture and assess those current pilots to apply emerging technology—especially within AI (complex information processing) and DeSci (automated governance, auditing, and/or incentivization) as umbrella categories for these efforts—and place them in the context of a broader framework of what we are trying to achieve with biomedical research.
d) Conceptualize gaps in our current efforts along with bridges from the current *status quo* to the desired future that may give us a better chance of success at transformational change to the systems of biomedical research and knowledge translation.
e) Communicate all aspects of the above areas in appropriate venues of biology, medicine, technology, and policy. This includes formal submissions of manuscripts on any related topics to this journal section and its partnered sections as appropriate.

This proposed conceptual framework is merely a jumping-off point for a broader consideration of how to maintain the core elements of the trust we have imbued in biomedical research as we continue to explore applications of emerging technology to improve its quality, manage its costs, and accelerate its contribution to the health and wellbeing of everyone. In the not-so-distant future, it is conceivable that we may be able to make all available relevant data on a topic or a patient accessible to any researcher to make AI-augmented and blockchain-audited hypothesis testing to provide near real-time, peer-validated contributions to evidence-based medicine. This could allow clinicians to query and access this near real-time evidence as part of compressing the 17 years it takes to go from bench to bedside by a factor of 10,000x—giving us new, actionable evidence-based precision medicine for patients in under a day. This future is within reach. Aligning behind a shared framework like *Scientia Machina* can bring it into our reality even faster. Better science. Cheaper research. Faster miracles.
